# Prevalence of serum MOG antibody and AQP4 antibody in optic neuritis after SARS-CoV-2 infection

**DOI:** 10.3389/fimmu.2023.1296518

**Published:** 2023-11-20

**Authors:** Chuan-bin Sun

**Affiliations:** Eye Center, Second Affiliated Hospital of Zhejiang University School of Medicine, Hangzhou, China

**Keywords:** optic neuritis, severe acute respiratory syndrome coronavirus 2, coronavirus disease 2019, aquaporin-4 antibody, myelin oligodendrocyte glycoprotein antibody

## Abstract

**Purpose:**

To evaluate the prevalence of serum myelin oligodendrocyte glycoprotein antibody (MOG-Ab) and aquaporin-4 antibody (AQP4-Ab) in optic neuritis (ON) patients after severe acute respiratory syndrome coronavirus 2 (SARS-CoV-2) infection by cell-based indirect immunofluorescence assay (CBA).

**Methods:**

In this prospective case series study, 35 patients clinically diagnosed as ON and laboratory-confirmed SARS-CoV-2 infection from 8 December 2022 to 8 February 2023 were included. All patients’ clinical and laboratory data were collected and analyzed.

**Results:**

The mean age of the 35 patients (46 eyes) was 38.2 years (ranging from 6 to 69 years), and 17 cases were female patients. Thirty-three and two cases showed positive SARS-CoV-2 RNA test results before or shortly after ON onset, respectively. ON occurred unilaterally in 24 cases and bilaterally in 11 cases. Ophthalmic examination revealed swollen optic disc in 37 eyes, normal optic disc in 6 eyes, and temporally or wholly paled optic disc in 3 eyes. CBA revealed seropositive MOG-Ab in 10 cases and AQP4-Ab in 2 cases, respectively, of which 2 AQP4-Ab-seropositive cases and 1 MOG-Ab-seropositive case had a past medical history of ON. Most ON patients showed a rapid and dramatic response to pulse steroid therapy. The median of BCVA at the onset and at the last follow-up was 20/500 (ranging from light perception to 20/20) and 20/67 (ranging from counting fingers to 20/20), respectively.

**Conclusion:**

Serum MOG-Ab and AQP4-Ab were detected in 28.6% (10/35) and 5.7% (2/35) ON cases after SARS-CoV-2 infection. SARS-CoV-2 infection may trigger an onset or a relapse of ON, as well as the production of MOG-Ab.

## 1 Introduction

Optic neuritis (ON) is an inflammatory, demyelinating ocular disease characterized by acute, painful visual loss (1–3). Currently, ON is believed to be an immune-mediated inflammation. However, most ON cases are idiopathic, and the exact etiology and targeted antigens of ON are still not known. Hence, ON is a clinical diagnosis based on medical history, ophthalmic examinations, and laboratory and MRI tests (1–4).

It is reported that the infection of many viruses such as varicella-zoster and herpes simplex virus can trigger a post-infectious immune-mediated ON or even the production of causative ON biomarkers such as myelin oligodendrocyte glycoprotein antibody (MOG-Ab) and aquaporin-4 antibody (AQP4-Ab) (1–4). Recently, dozens of ON were reported in patients after severe acute respiratory syndrome coronavirus 2 (SARS-CoV-2) infection since the coronavirus disease 2019 (COVID-19) worldwide pandemic from December 2019 (5–13). Moreover, seropositive MOG-Ab and AQP4-Ab were reported in ten and two ON cases after SARS-CoV-2 infection, respectively, which indicated that SARS-CoV-2 infection may trigger an onset of ON or even the production of MOG-Ab and AQP4-Ab (5–13).

Currently, the prevalence of serum AQP4-Ab and MOG-Ab in ON cases after SARS-CoV-2 infection is still not clear because of only a small number of cases reported in the literature (5–13). On 7 December 2022, the termination of the dynamic zero COVID-19 strategy by the Chinese government immediately initiated a resurgence of the COVID-19 pandemic throughout China (14, 15). Then, complaints of sudden visual loss and subsequent clinical diagnosis of ON dramatically occurred in patients after SARS-CoV-2 infection in ophthalmology clinics throughout China, providing us a chance to investigate the prevalence of serum AQP4-Ab and MOG-Ab in ON cases after SARS-CoV-2 infection.

## 2 Patients and methods

### 2.1 Patients

In this prospective case series study, 35 patients clinically diagnosed as ON after SARS-CoV-2 infection from 8 December 2022 to 8 February 2023 at the Neuro-ophthalmology Clinic of the Second Affiliated Hospital of Zhejiang University School of Medicine were included. Detailed medical records including medical history, complete ophthalmic examination, color fundus photography, visual field test, orbital or cranial MRI examination, and serum testing data were collected and analyzed. This study was conducted according to the tenets of the Declaration of Helsinki. Informed consent was obtained from all patients. Institutional review board approval was obtained from the Second Affiliated Hospital of Zhejiang University School of Medicine.

The diagnosis of ON is based on its typical ophthalmic manifestations: 1) acute or subacute visual loss; 2) defected direct pupillary light reflex; 3) swollen or normal optic disc; 4) typical visual field defects of ON including large central scotoma, generalized depression, arcuate scotoma, ring scotoma, or tunnel vision; and 5) exclusion of other optic neuropathies including ischemic, traumatic, compressive, toxic, or hereditary optic neuropathies.

The inclusion criteria were as follows: 1) definite ON diagnosis and 2) positive SARS-CoV-2 RNA test results by reverse transcription‐polymerase chain reaction assay from a nasopharyngeal swab sampled from patients prior to or shortly after the onset of ON. The exclusion criteria were as follows: 1) positive serum IgM or IgG antibodies of other pathogens including *Treponema pallidum*, *Mycobacterium tuberculosis*, herpes viruses, hepatitis viruses, or HIV and 2) other ocular diseases including severe cataract, glaucoma, or other optic neuropathies which may influence the observation and evaluation of ON.

### 2.2 Ophthalmic examination and MRI test

All patients underwent best corrected visual acuity (BCVA), slit-lamp examination and direct ophthalmoscopy, color fundus photography, and visual field test. Color fundus photography was taken using Canon CX-1 (Canon Company, Japan), the visual field was tested using the 30 program for Octopus 900 (Haag-Streit Diagnostics, Switzerland) perimeter, and low vision program was used for patients with BCVA lower than 20/200 but better than hand motion.

Orbital or cranial MRI examination was tested using T2-weighted imaging sequence with fat suppression and fluid-attenuated inversion recovery sequence and T1-weighted imaging sequence with fat suppression sequence and gadolinium enhancement.

### 2.3 Serum testing

All patients underwent serum tests listed as follows: serum IgM or IgG antibodies to pathogens including *T. pallidum*, *M. tuberculosis*, herpes viruses, hepatitis viruses, and HIV, as well as antinuclear antibodies including antinuclear antibody, anti-double-stranded DNA, anti-Sjogren syndrome A or Sjogren syndrome B, and anticardiolipin antibodies.

Serum AQP4-Ab and MOG-Ab sampled from patients at or shortly after the onset of ON were commercially tested (Aegicare, China) by CBA using HEK-293 cells overexpressing AQP-4 M1 isoform or full-length MOG on their cellular membranes, respectively, according to the procedures described in previous reports (16, 17).

### 2.4 Therapy

All 35 patients were treated with intravenous methylprednisolone 500 mg (or 1,000 mg) per day for 3 or 5 days based on the severity of vision loss and followed by tapered oral prednisone therapy. For ON cases dual negative for AQP4-Ab and MOG-Ab, steroid therapy followed the Optic Neuritis Treatment Trial therapy suggestions. For ON cases with AQP4-Ab or MOG-Ab positive, steroid (and subsequent oral immunosuppressive agent) therapy followed the guidelines for the therapy of AQP4-Ab- or MOG-Ab-positive ON.

## 3 Results

### 3.1 Serum test of MOG-Ab and AQP4-Ab in ON patients after SARS-CoV-2 infection

The CBA test revealed MOG-Ab positive in 10 cases and AQP4-Ab positive in 2 cases, respectively, as well as MOG-Ab and AQP4-Ab dual negative in the other 21 cases (**Table 1**). One AQP4-Ab-positive case had a past medical history of ON in the ipsilateral eye, whereas one AQP4-Ab-positive and one MOG-Ab-positive case had a past medical history of ON in the contralateral eye.

**Table 1:** Demographic characteristics and serum test results of optic neuritis after SARS-CoV-2 infection.

No.	Gender	Age (years)	Eye	PMH	CBA test	Other autoantibodies
1	Female	67	OD	None	Negative	Negative
2	Female	46	OD	None	MOG-Ab 1:100	Negative
3	Male	44	OD	None	Negative	Negative
4	Female	6	OU	None	AQP-Ab 1:320	Negative
5	Male	46	OU	None	MOG-Ab 1:10	Negative
6	Male	41	OD	None	Negative	Negative
7	Male	56	OD	ON in OS	Negative	Anti-CL IgM++
8	Female	26	OD	None	Negative	Negative
9	Female	35	OS	None	Negative	ANA+, anti-Ro-52+, anti-β_2_-GP I IgM++
10	Female	40	OS	None	Negative	Negative
11	Male	23	OU	None	Negative	Negative
12	Female	62	OU	None	Negative	Negative
13	Male	11	OS	None	Negative	Negative
14	Female	35	OS	None	Negative	Negative
15	Male	30	OD	None	MOG-Ab 1:100	Negative
16	Male	44	OU	None	MOG-Ab 1:320	Negative
17	Male	49	OS	None	Negative	Negative
18	Male	23	OD	None	Negative	Negative
19	Male	43	OU	None	Negative	Negative
20	Female	56	OS	ON in OU	Negative	Negative
21	Male	17	OD	None	MOG-Ab 1:320	Negative
22	Male	44	OS	None	Negative	Negative
23	Female	53	OD	ON in OS	MOG-Ab 1:100	Anti-CL IgM++, anti-β_2_-GP I IgM++
24	Female	56	OD	None	MOG-Ab 1:32	Negative
25	Female	31	OS	ON in OD	AQP-Ab 1:320	Negative
26	Female	23	OS	None	Negative	Negative
27	Male	37	OU	None	MOG-Ab 1:32	Negative
28	Female	38	OU	None	Negative	Negative
29	Male	20	OU	None	Negative	Negative
30	Female	27	OD	None	MOG-Ab 1:100	Negative
31	Female	53	OS	None	Negative	ANA+, anti-SSA+, anti-β_2_-GP I IgM++
32	Female	36	OS	ON in OS	Negative	Negative
33	Male	6	OU	None	MOG-Ab 1:320	Negative
34	Male	69	OU	None	Negative	Negative
35	Male	43	OD	ON in OD	Negative	Negative

PMH, post-medical history; CBA, cell-based assay; ON, optic neuritis; OS, left eye; OD, right eye; OU: both eyes; MOG-Ab, oligodendrocyte glycoprotein antibody; AQP-Ab, aquaporin-4 antibody; ANA, antinuclear antibody; Anti-CL, anticardiolipin antibody; anti-SSA, anti-Sjogren syndrome A antibody; anti-β_2_-GP I, anti-β_2_-glycoprotein I antibody.

Anti-β_2_-glycoprotein I IgM antibody, anticardiolipin IgM antibody, antinuclear antibody, anti-Ro-52 antibody, and anti-Sjogren syndrome A antibody were detected positive in 3 cases, 2 cases, 2 cases, 1 case, and 1 case, respectively (**Table 1**). All 35 cases showed negative test results for IgM or IgG antibodies of pathogens including *Treponema*, *M. tuberculosis*, herpes viruses, hepatitis viruses, or HIV.

### 3.2 Domestic and systemic characteristics of ON after SARS-CoV-2 infection

Thirty-five patients (46 eyes) with confirmed diagnosis of ON and SARS-CoV-2 infection were included in this study. The mean age of the patients was 38.2 years (ranged from 6 to 69 years), and 17 cases were female patients. All 35 cases got at least two shots of free inactivated vaccine (Sinovac Life Sciences Company or China National Biotec Group Company, details not available) before the resurgence of COVID-19 in December 2023 in China.

Six of the 35 ON cases had a past medical history of ON, of which three AQP4-Ab-positive ON cases were still under low-dose prednisone or an oral immunosuppressive agent (azathioprine) therapy and the other three ON cases were dual negative for AQP4-Ab and MOG-Ab and already stopped medical therapy for months or years before the onset of ON. Another case had a medical history of rectal carcinoma with hepatic and pulmonary metastasis and was still under antitumor agent therapy.

Thirty-three and two cases showed positive SARS-CoV-2 RNA test results before or shortly after ON onset, respectively. Thirty-one of the 35 patients showed systemic symptoms including fever (27/31), cough (6/31), myalgias (4/31), headache (3/31), and laryngalgia (1/31). The other four cases had no systemic symptoms although the SARS-CoV-2 RNA tests were positive prior to ON onset. The mean interval between the onset of systemic symptoms and visual loss was 12.3 days in 29 cases (ranging from 2 to 51 days) with systemic symptoms occurring prior to ON onset and 11.5 days in 2 cases (3 days in 1 case and 20 days in the other) with systemic symptoms occurring after ON onset.

### 3.3 Ophthalmic and MRI characteristics of ON after SARS-CoV-2 infection

**Figure 1:**
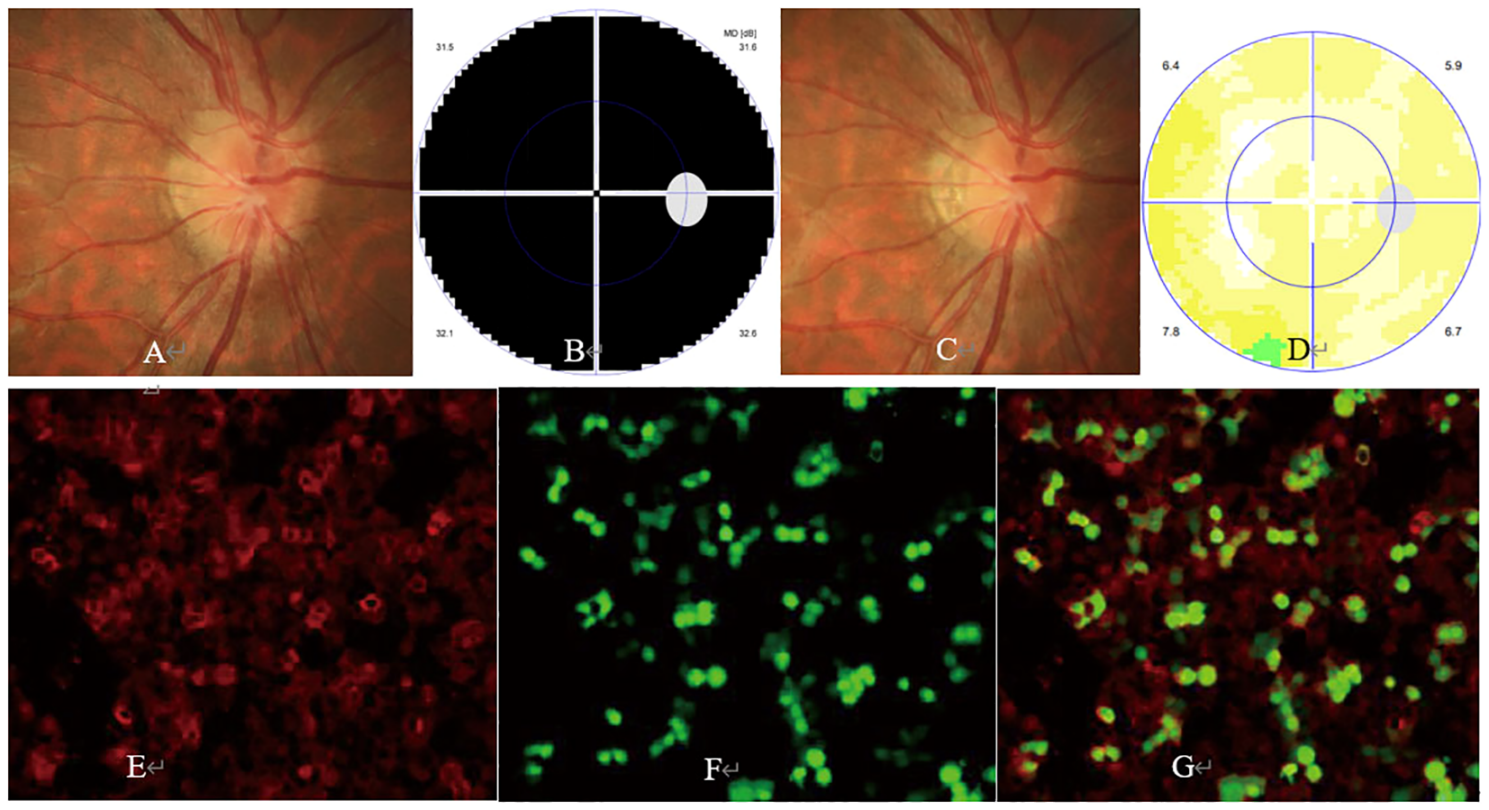
Ophthalmic examination and cell-based assay (CBA) in a myelin oligodendrocyte glycoprotein antibody (MOG-Ab)-positive optic neuritis (ON) case. The patient was a 30-year-old man with a complaint of sudden visual loss in the right eye. At presentation, his right eye had a visual acuity of hand motion, with a swollen optic disc **(A)** and generalized depression in the visual field test **(B)**. Five days after steroid pulse therapy, his vision in the right eye improved to 20/20, with a relieved swollen optic disc **(C)** and normal visual field **(D)**. CBA showed a positive test result for MOG-Ab with a titer of 1:100 [**(E)** MOG-Ab staining, **(F)** MOG protein staining, **(G)** merged image].

**Figure 2:**
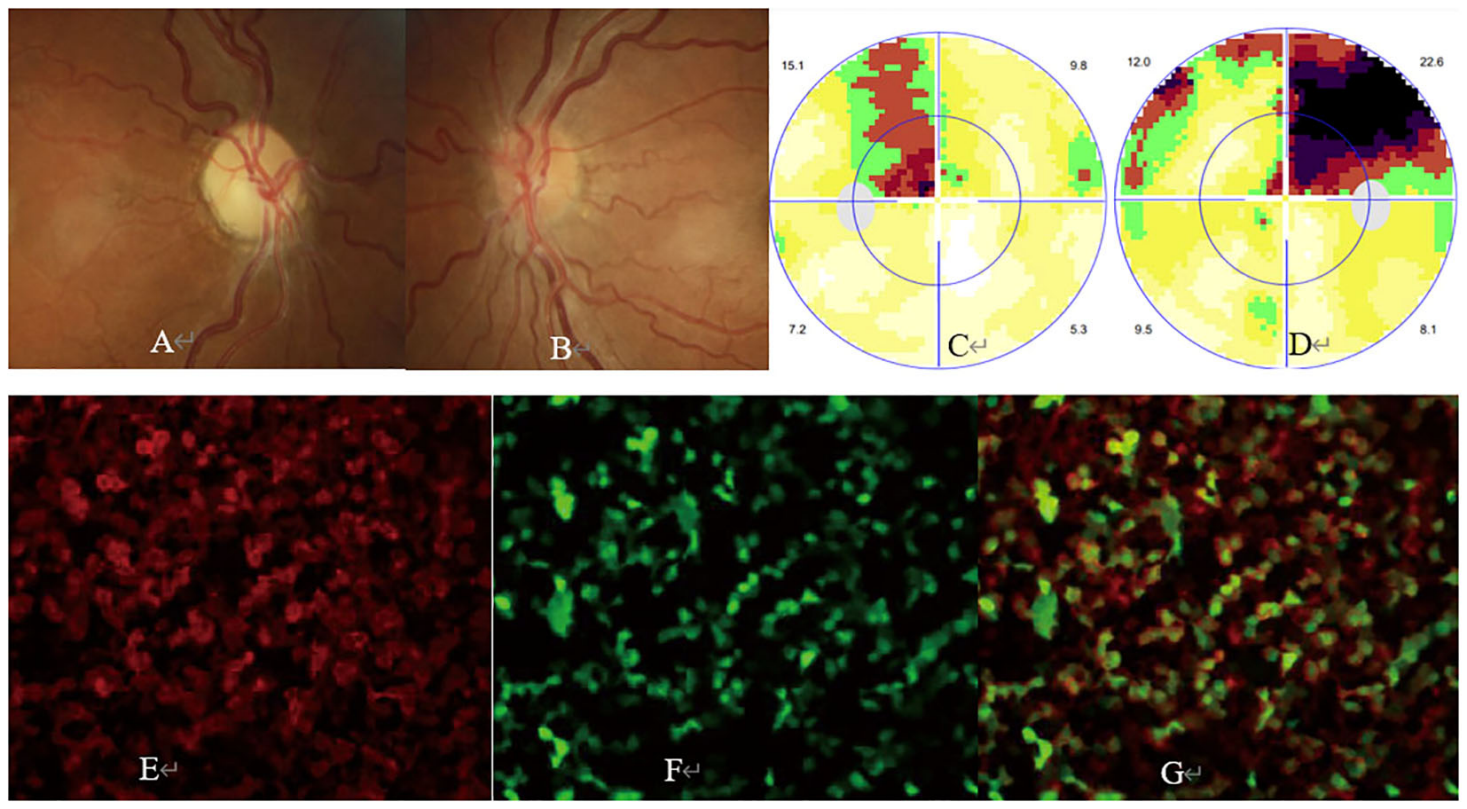
Ophthalmic examination and CBA in an aquaporin-4 antibody (AQP4-Ab)-positive ON case. The patient was a 31-year-old woman with a complaint of sudden visual loss in the left eye. She had a past medical history of AQP4-Ab-positive ON in the right eye 1 year ago and was still under mycophenolate mofetil therapy before the onset of ON in the left eye. At presentation, ophthalmic examination showed visual acuity of 20/67 and 20/200 and pale **(A)** and slightly swollen optic disc **(B)** in the right and left eye, respectively. The visual field test showed a large centrocecal scotoma in the left **(C)** and right **(D)** eye, respectively. CBA showed a positive test result for AQP4-Ab with a titer of 1:320 [**(E)** AQP4-Ab staining, **(F)** AQP-4 protein staining, **(G)** merged image].

**Figure 3:**
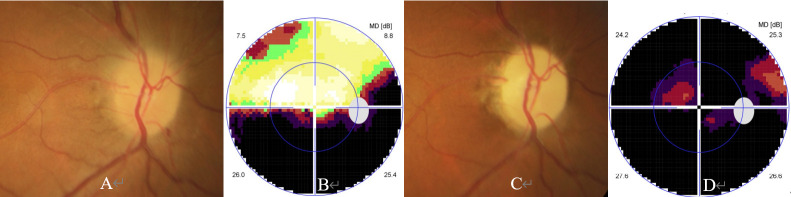
Ophthalmic examination and CBA in an AQP4-Ab and MOG-Ab dual negative ON case. The patient was a 67-year-old woman with a complaint of reoccurred visual loss in the right eye. She experienced sudden visual loss in the same eye 4 months ago, showed partial response to steroid pulse therapy, and stopped steroid therapy 1 month ago. Her ophthalmic examination performed 4 months ago showed visual acuity of 20/40, a swollen optic disc **(A)**, and inferior horizontal hemianopia **(B)** in the visual field test. At presentation, ophthalmic examination showed visual acuity of hand motion, a pale optic disc **(C)**, and generalized depression in the visual field test **(D)** in the right eye.

ON occurred unilaterally in 24 cases and bilaterally in 11 cases. At the onset, BCVA of all 46 ON eyes ranged from light perception to 20/20 with a median of 20/500. Ophthalmic examination revealed swollen optic (**Figures 1A, C**, **2B**, **3A**) in 37 eyes, normal optic disc in 6 eyes, and temporally or wholly paled optic disc (**Figures 2A**, **3C**) in 3 eyes. The visual field test was performed in 43 eyes and showed generalized depression (**Figures 1B**, **3D**), large central/centrocecal scotoma (**Figures 2C, D**), tunnel vision, arcuate scotoma, and horizontal/vertical hemianopsia (**Figure 3B**) in 17, 10, 7, 7, and 2 cases, respectively. Typical ophthalmic manifestations of ON cases with MOG-Ab positive, with AQP-4Ab positive, and with MOG-Ab and AQP-4Ab duonegative were shown in **Figures 1A–G**, **2A–G**, **3A–D**, respectively. Orbital or cranial MRI was examined in 29 cases and revealed an enlarged optic nerve with contrast enhancement in 15 cases and a normal optic nerve in 14 cases. No brain lesions were found in all the cases. No imaging signs of tumor cell infiltration in the optic nerves were found in the patients concurrent with rectal carcinoma and hepatic and pulmonary metastasis.

The mean follow-up was 7.6 weeks ranging from 8 to 16 weeks. Most ON patients showed a rapid and dramatic response to pulse steroid therapy and subsequent oral prednisone. At the last follow-up, BCVA of all 46 ON eyes ranged from counting fingers to 20/20 with a median of 20/67. Thirty-one of the 46 ON eyes got a visual improvement of at least 0.3 LogMAR (15 letters in the EDTRS chart), and 23 of the 46 ON eyes had a BCVA of at least 20/67.

## 4 Discussion

Previous investigations have revealed that many pathogens including viruses, *Mycoplasma*, and *M. tuberculosis* can trigger an immune-mediated ON and induce the production of MOG-Ab and AQP4-Ab (1–4). Although ocular involvement including conjunctivitis, uveitis, retinal hemorrhage, and vascular occlusion is not uncommon in COVID-19 cases, optic neuropathies especially ON are rarely reported after SARS-CoV-2 infection (18–23).

The termination of the dynamic zero COVID-19 strategy and the subsequent resurgence of COVID-19 in China led to a marked increase in ON and provided us a chance to investigate the prevalence of serum AQP4-Ab and MOG-Ab in ON patients after SARS-CoV-2 infection. In this study, we included 35 ON cases with confirmed SARS-CoV-2 infection, which, to the best of our knowledge, is the largest case series study until now. Our study revealed that 29 of 35 cases manifested as an initial onset of ON, and the other 6 cases presented as a relapse of ON after SARS-CoV-2 infection, which indicated that SARS-CoV-2 infection can trigger an initial onset or a relapse of ON (24).

In this study, serum MOG-Ab and AQP4-Ab were detected positive in 10 and 2 cases, respectively. Although 3 of the above 12 cases had at least one episode of ON before the SARS-CoV-2 infection, i.e., 2 AQP4-Ab-positive and 1 MOG-Ab-positive ON case had a past medical history of ON, our study indicated that MOG-Ab ON was probably triggered after SARS-CoV-2 infection in at least 9 cases. This finding gives further support that SARS-CoV-2 infection may trigger an onset of MOG-Ab-positive ON and the production of MOG-Ab (5, 8–13).

Our study, consistent with previous investigations, indicated that SARS-CoV-2 infection may trigger the production of MOG-Ab and the onset of an immune-mediated ON in COVID-19 cases (5, 8–13). The probable mechanism of MOG-Ab production in patients after SARS-CoV-2 infection is molecular mimicry, i.e., SARS-CoV-2 may express an antigen mimicking the MOG protein expressed in the membrane of astrocytes (5, 8–13). Another less likely mechanism of MOG-Ab production is an opportunistic exposure of the MOG protein to antigen-presenting cells during SARS-CoV-2-induced inflammation in the white matter of the central nervous system or in the optic nerve. The mean interval of 12.3 days between the onset of SARS-CoV-2 infection and ON and the immediate, dramatic response to pulse steroid therapy without antiviral agents in most ON cases in this study propose that ON after SARS-CoV-2 infection is an immune-mediated and not a direct infectious inflammation. Similar findings were reported in previous literature. Moreover, SARS-CoV-2 infection may disrupt and increase the permeability of the blood–brain barrier by increasing the expression of inflammatory cytokines and then trigger a relapse of ON.

In summary, our study indicates that SARS-CoV-2 infection may trigger an initial onset or a relapse of ON and the production of MOG-Ab. ON after SARS-CoV-2 infection shows a good response to pulse steroid therapy. All these findings further support that SARS-CoV-2 infection-related ON is a post-infectious immune-mediated inflammation. However, our study still has some limitations; for example, CBA is not concurrently tested in CSF samples of ON patients due to the patients’ consent and financial consideration. Hence, some dual negative test results for AQP4-Ab and MOG-Ab may be false-negative since previous investigations revealed that AQP4-Ab can be detected positive in CSF samples of some AQP4-Ab-seronegative neuromyelitis optica patients. Moreover, the direct relationship between SARS-CoV-2 infection and MOG-Ab production needs more cellular and animal experiments to prove and confirm.

## Data availability statement

The original contributions presented in the study are included in the article/supplementary material. Further inquiries can be directed to the corresponding author.

## Ethics statement

The studies involving humans were approved by the Second Affiliated Hospital of Zhejiang University School of Medicine. The studies were conducted in accordance with the local legislation and institutional requirements. The participants provided their written informed consent to participate in this study.

## Author contributions

CS: Conceptualization, Data curation, Investigation, Methodology, Writing – original draft, Writing – review & editing.
